# The safety and efficacy of additional chest tube placement in patients with prolonged air leaks after pulmonary resection: a propensity score-matched analysis

**DOI:** 10.3389/fmed.2024.1484327

**Published:** 2024-10-08

**Authors:** Qingwang Hua, Suyue Liu, Lu Shen, Zhenhua Yang, Haibo Shen

**Affiliations:** Department of Thoracic Surgery, Ningbo No.2 Hospital, Ningbo, China

**Keywords:** air leak, chest drains, postoperative, pulmonary surgery, tube

## Abstract

**Background:**

This study evaluates the symptomatic management of prolonged pleural air leaks following pulmonary resection, assesses the efficacy and safety of chest tube placement, and introduces experiences with high-positioned chest tube insertion.

**Methods:**

We retrospectively reviewed 84 patients with prolonged pleural air leaks after lung surgery at Ningbo No.2 Hospital from January 2022 to December 2023. These patients were divided into a conservative treatment group (Group A, *n* = 64) and a chest tube placement group (Group B, *n* = 20). The propensity score matching method was applied to balance confounders between the two groups, resulting in 12 matched pairs. The study compared the time to chest tube removal, average hospital stays time, postoperative drainage volume, and facial visual analog pain score between the two groups.

**Results:**

The average hospital stays and chest tube removal time of patients in group B were significantly lower than those of patients in group A (8.00 ± 1.12 vs. 9.75 ± 1.60 days, *P* = 0.003, 6.92 ± 1.08 vs. 8.58 ± 1.67 days, *P* = 0.005, respectively). However, the mean facial visual analog pain score in group B was higher than that in group A (1.58 ± 0.58 vs. 1.00 ± 0.01, *P* = 0.020). There were no significant differences between the two groups in terms of postoperative drainage volume.

**Conclusions:**

For patients with prolonged air leaks, additional chest tube placement postoperatively significantly reduces both hospitals stay duration and chest tube indwelling time compared to conservative treatment. This method may be a potential treatment measure for prolonged air leak in selected patients.

## Introduction

Non-small cell lung cancer (NSCLC) has emerged as a predominant malignancy with significant morbidity and mortality rates globally (1). In China, the incidence and prevalence of NSCLC have been steadily rising, posing a substantial public health challenge (2). The increasing burden of NSCLC underscores the necessity for effective surgical interventions and postoperative care strategies to enhance patient outcomes and mitigate the adverse effects associated with this condition.

The surgical treatment of NSCLC has advanced rapidly, with video-assisted thoracoscopic surgery (VATS) now being the predominant approach (3). Despite its minimally invasive nature and quicker recovery times, uniportal VATS is also frequently accompanied by various postoperative complications, which remain a focal point of clinical research aimed at improving surgical outcomes (4). Among these complications, prolonged air leak (PAL) being one of the most common issues, stand out due to its impact on patient recovery (5–7). PAL is characterized by the inability to remove the chest tube postoperatively, resulting in extended hospital stays, which contradicts the principles of enhanced recovery after surgery (ERAS) (8).

The etiology of PAL is multifactorial, and its management focuses on promoting rapid lung re-expansion and effective drainage of the residual pleural space. Conventional conservative treatments for PAL include external negative pressure drainage, pleurodesis, and endoscopic treatments, etc. However, these approaches are often ineffective and costly in certain situations and fail to significantly reduce hospital stay, which may increase morbidity and mortality after lung resection and hinder the recovery process (9).

In this study, we conducted a retrospective analysis to evaluate the effectiveness of bedside chest tube placement in patients with prolonged air leaks following pulmonary resection. Our findings suggest that this intervention can substantially enhance lung re-expansion and reduce hospital stay durations, offering a more efficacious and inexpensive approach compared to traditional conservative treatments. This investigation highlights the importance of re-evaluating current PAL management protocols and emphasizes the potential benefits of incorporating timely chest tube placement into postoperative care strategies.

## Methods

### Patient inclusion and exclusion criteria

A retrospective analysis was conducted on patients who underwent video-assisted thoracoscopic surgery (VATS) for lung cancer at our institution (Department of Thoracic Surgery, Ningbo No.2 Hospital) from January 01 2022 to December 31 2023. Inclusion criteria were as follows: (I) All patients underwent preoperative enhanced chest CT scans to determine clinical staging, and the surgeries were all VATS procedures. Intraoperative frozen section analysis was conducted by pathology experts to confirm a pathological diagnosis of non-small cell lung cancer; (II) All patients' baseline vital conditions were thoroughly evaluated preoperatively to ensure they could tolerate lung cancer surgery; (III) No cardiovascular or cerebrovascular infarcts occurred within 3 months prior to surgery; (IV) Normal coagulation function with no use of anticoagulants in the 2 weeks preceding surgery. (V) Prolonged air leak while Chest tube indwelling for more than 5 days.

**Figure 1:**
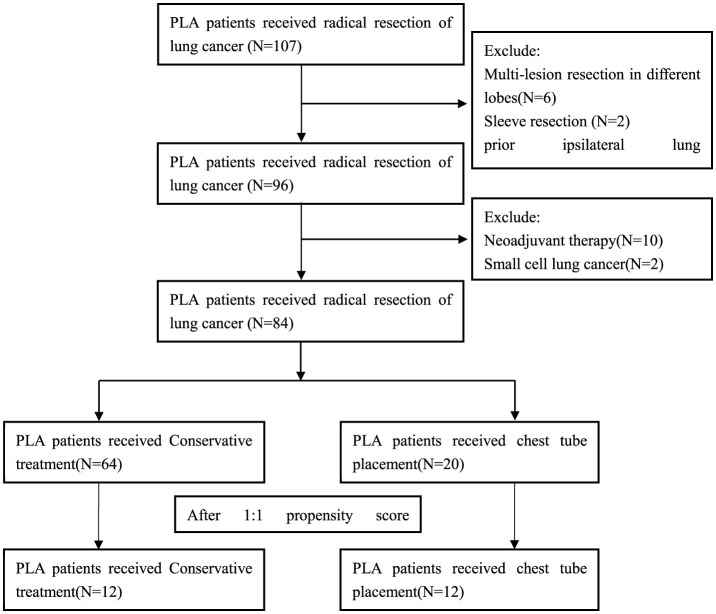
Flow chart for inclusion and exclusion of patients.

Exclusion criteria were as follows: (I) Multi-lesion resection in different lobes or Sleeve bronchial resection; (II) VATS converted to thoracotomy patients. (III) Prior ipsilateral lung surgery; (IV) Neoadjuvant therapy patients; (V) Pathological diagnosis was small cell lung cancer; (VI) Postoperative bronchoscopy revealed bronchopleural fistula. The flow chart for inclusion and exclusion of patients as Figure 1. This study was conducted in accordance with the Helsinki Declaration (revised in 2013). The study was approved by ethics board of Ningbo No.2 Hospital and informed consent was obtained for each patient.

### Grouping criteria and chest tube insertion/removal criteria

**Figure 2:**
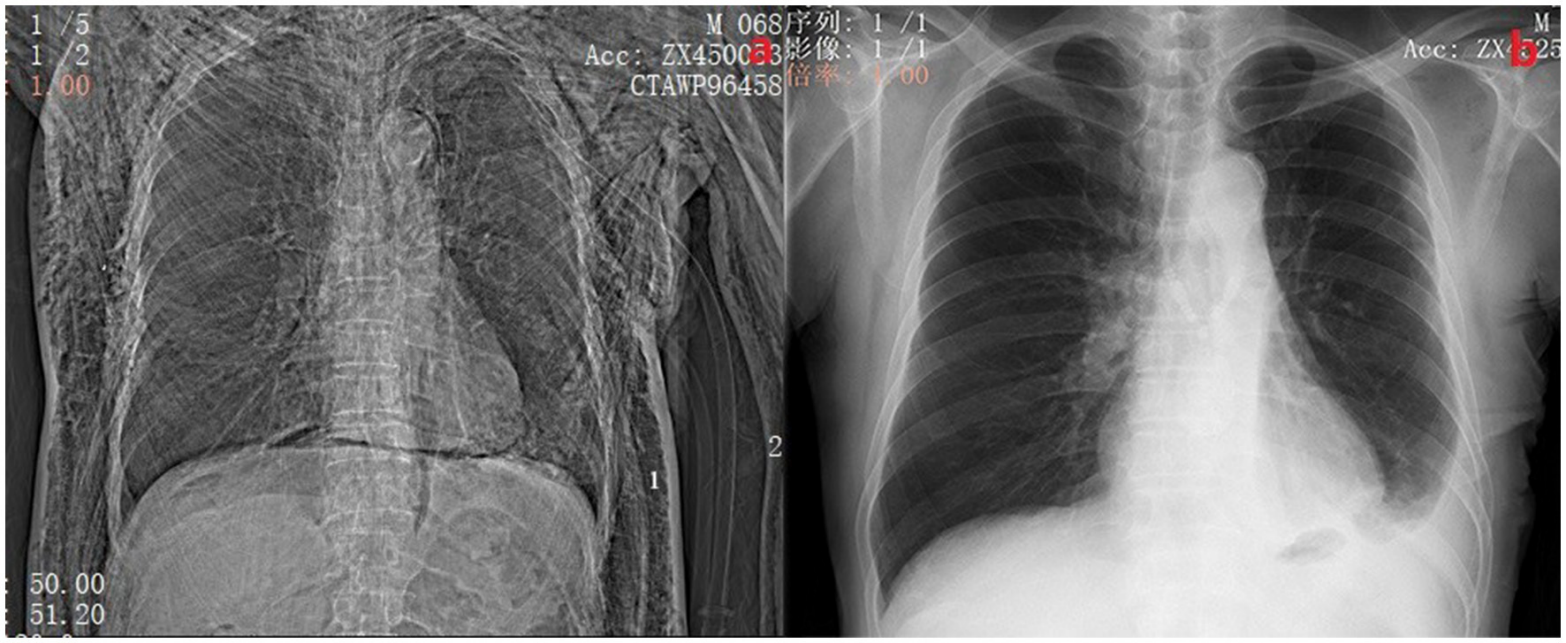
**(A)** Shows a patient with postoperative pneumothorax and subcutaneous emphysema with 2 chest tubes inserted. The side marked with 1 is the chest tube placed during surgery, and the side marked with 2 is the chest tube placed at the bedside. **(B)** Shows a chest X-ray of the same patient 2 weeks after discharge.

When the air leak lasted for more than 5 days, we defined the patient as having PAL (10). We divided these PAL patients into group A and group B according to whether the patients had another chest tube inserted. If the patients had another chest tube inserted, they belonged to group B. Otherwise, they belonged to group A. The conservative treatment group (Group A) received extracorporeal negative pressure drainage (-8-−10 cm H_2_O) and pleurodesis, such as intrathoracic injection of diluted povidone-iodine or 50% glucose solution, autologous blood pleurodesis. This treatment should be considered first for patients with PLA. In general, patients in group A presented with simple air leak without other clinical symptoms. Their chest x-ray usually shows a pneumothorax volume of < 30%. While for the chest tube placement group (Group B), we inserted another chest tube in the pleural cavity. The chest tube was placed in the following situations: (I) pneumothorax volume ≥30% after conservative treatment; (II) pneumothorax with extensive subcutaneous emphysema (Figure 2a); (III) the grade of air leak does not decrease after conservative treatment. The drainage status of all chest tubes will be included in the observation regardless of group A or group B. If there is no air leakage within 24 h and the total amount of light limpid fluid drainage in 24 h is ≤ 300 mL, the chest tube removal should be considered.

### Surgical information and follow-up indicators

All patients underwent uniportal VATS procedure and were performed by the same surgical team. All patients received standardized pulmonary resection and mediastinal lymphadenectomy according to the Chinese Medical Association guidelines for the clinical diagnosis and treatment of lung cancer (edition 2018). The incision hole was located at the anterior axillary line of the 4th or 5th interspace for pulmonary resection (including lobectomy and Sub-lobar resection) and lymphadenectomy with a length of 3–4 cm. Generally, the 4th interspace for right lobes and the 5th interspace for left lobes. At the end of surgery, the conventional chest tube (24 Fr, 8.00 mm in diameter) was used. The chest tube was inserted from the incision straight to the top of the chest through the anterior mediastinum pathway, which was connected to water-seal bottles without negative pressure. After the operations, we collected the postoperative thoracic drainage volume, the average VAS pain scores in incisions, average hospital stay time, chest tube removal time during the perioperative period, levels of serum CRP and pulmonary complications during hospitalization. If the chest X-ray or CT shows that the residual lung re-expansion and without obvious inflammation (Reference the levels of serum C-reactive protein (CRP) and bodies' temperature), and the patient with no obvious complaints of discomfort, then the patient was acceptable criteria for discharge (Figure 2b). 2 weeks after discharge, the incision sutures were removed and chest X-rays were reviewed. A follow-up chest CT scan was also performed 1 month after discharge to ensure the patient's recovery.

### High position chest tube insertion procedure

**Figure 3:**
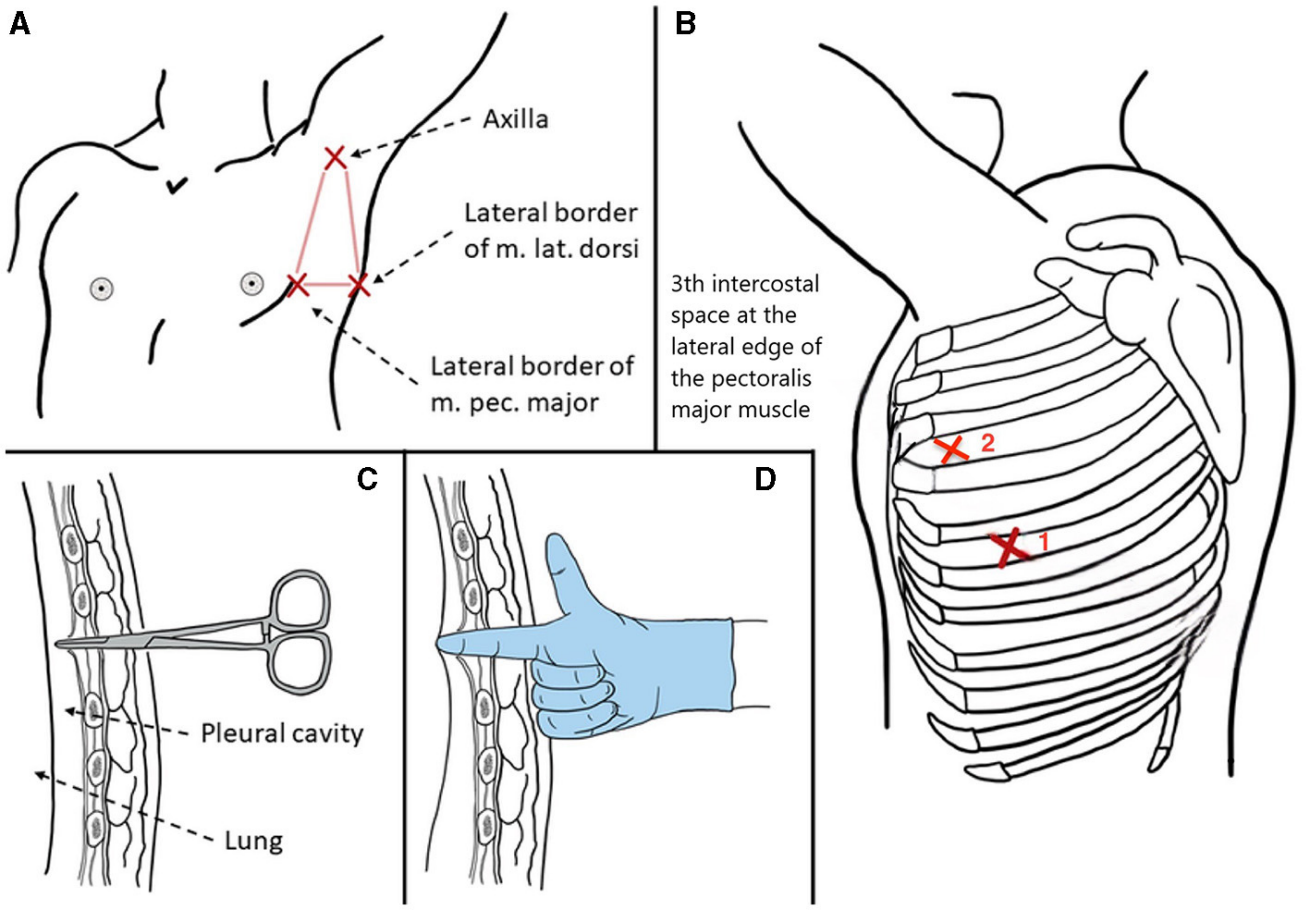
The high position chest tube insertion procedure.

**Figure 4:**
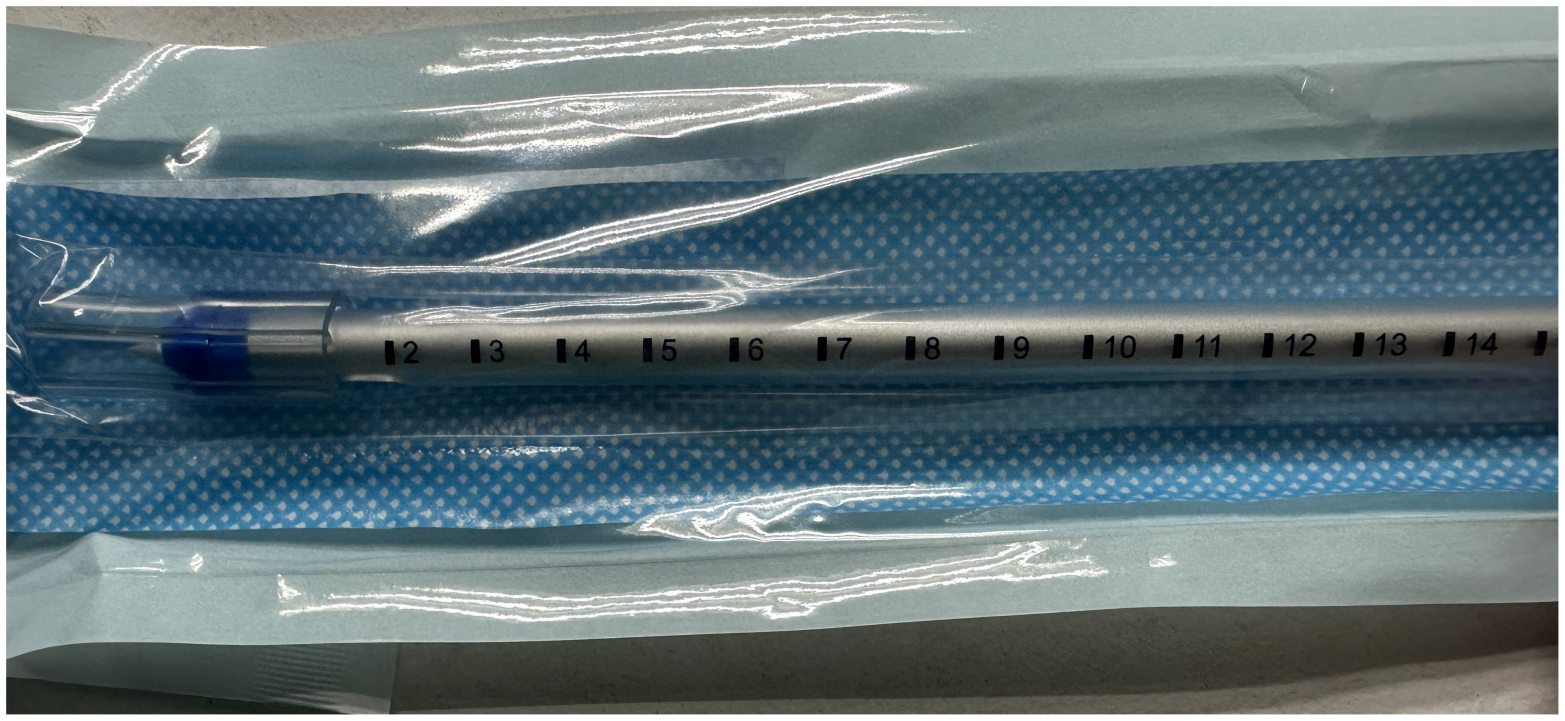
The chest tube with a needle.

The chest tubes were performed by the same person. Generally, we placed the chest tube with a needle (PAHSCO, 28 Fr with 9.33 mm in diameter, Figure 4) in the “triangle of safety,” which was inserted straight to the top of the chest cavity through the anterior mediastinum pathway, the key points of simple procedures: (a) Usually, the patient lies in a reclining position on the bed with the arms abducted, identify the “triangle of safety:” the center of the axilla, the lateral aspect of musculus latissimus dorsi, and the lateral pectoralis major at the line of the nipple. (b) An incision approximately with 1–2 cm was made at the 3th intercostal space at the lateral edge of the pectoralis major muscle nearby the anterior axillary line through the “triangle of safety,” as like red cross mark b-2. The b-1 red cross mark is the first chest tube which was placed after surgery. (c) Muscle tissue is then dissected using a blunt instrument e.g., a set of arterial forceps, creating a canal to the parietal pleura which is then breached for access to the pleural cavity. A hiss of air, or ooze of blood may present at this point. (d) The pleural cavity is explored using a finger, assessing for the position of the lung and any adhesions. The resulting tube remains open to allow enough air to enter the chest cavity. In the case of artificial pneumothorax, the chest tube is inserted into the chest cavity under the guidance of needle. Be careful not to puncture the lung or other tissues to avoid secondary damage. In our experience, the tube with the side hole is usually placed in the top of the chest cavity at a scale of 12–14 cm [Figure 3 (11)]. Moreover, the tube was connected to water-seal bottles with negative pressure.

### Data collection and statistical methods

All patients' data were collected from hospital charts or databases. SPSS 26.0 software was used to analyze the data (IBM SPSS Statistics, USA). Continuous variables were presented as means ± standard deviation or medians (range), and the comparison between the two groups was performed using the *t*-test. Categorical variables were presented as counts or rates and A chi squared test or Fisher s exact test was used to compare dichotomous variables. The Mann-Whitney *U*-test is used for statistical analysis of rank data. The balance of measured variables between groups after propensity score-matching was analyzed using a paired *t*-test for continuous measures and the McNemar test for categorical variables. Propensity score matching was used to mitigate discrepancies in the characteristics of the study cohort that may influence our outcomes. Cases were matched **1:1** with a caliper size of 0.02. Variables used for matching were age, gender, smoking history, COPD, FEV1, FEV1%, and the operation style. A *P*-value < 0.05 was considered statistically significant.

## Result

A total of 84 patients were enrolled in the study (Figure 1). Patients were classified into the conservative treatment group (Group A, *n* = 64) and the chest tube placement group (Group B, *n* = 20) according to the different treatment methods. After calculating the propensity scores (ratio = 1:1), 12 pairs were matched. Table 1 shows the patient demographic and clinical data before and after propensity score matching. The basic line of patients who undergoing chest tube placement were quite similar compared to those in the conservative treatment group except for the age, gender, COPD and pTNM stage (*P* < 0.05) before PSM matching (Table 1). After propensity score-matching, both groups were well-matched in theses parameters. Baseline characteristics of the matched patients were listed in Table 2. The mean operation time was 101.67 ± 11.46 min in group A and 98.33 ± 5.29 min in group B (*P* = 0.386). The mean Thoracic drainage volume in group A is less than that in group B (544.58 ± 242.44 ml vs. 897.50 ± 266.53 ml, *P* = 0.660), but without statistical differences, also as the parameter of Blood loss (*P* = 0.104), Pleural adhesion (*P* = 0.453) and the Serum C-reactive protein before discharge (39.80 ± 22.95 vs. 53.29 ± 28.23 mg/L, *P* = 0.660). The Average hospital stay and the Chest tube removal time in group A is significantly longer than those patients in group B (9.75 ± 1.60 vs. 8.00 ± 1.12 days, *P* = 0.003, 8.58 ± 1.67 vs. 6.92 ± 1.08 days, *P* = 0.005, respectively). However, the mean facial visual analog pain score in group A was lower than that in group B, which also indicated a significant difference (1.00 ± 0.01 vs. 1.58 ± 0.58, *P* = 0.020).

**Table 1:** Demographics and clinical data before PSM and after PSM.

**Variables**	**Before PSM**			**After PSM**		
	**Conservative treatment group** ***N*** **(%) (*****n*** = **64)**	**Chest tube group** ***N*** **(%) (*****n*** = **20)**	***P*** **-value**	**Conservative treatment group** ***N*** **(%) (*****n*** = **12)**	**Chest tube group** ***N*** **(%) (*****n*** = **12)**	***P*** **-value**
Age (years old)	61.69 ± 12.24	67.90 ± 7.67	0.036	64.83 ± 7.17	67.92 ± 9.61	0.249
Gender			0.005			1.000
Male	25 (39.1)	15 (75.0)		7 (58.3)	7 (58.3)	
Female	39 (60.9)	5 (25.0)		5 (41.7)	5 (41.7)	
**Past history**						
Smoking	27 (42.2)	12 (60.0)	0.163	7 (58.3)	7 (58.3)	1.000
Hypertension	36 (56.3)	16 (80.0)	0.056	8 (66.7)	9 (75)	1.000
Diabetes	10 (15.6)	6 (30.0)	0.153	1 (8.3)	2 (16.7)	1.000
COPD	4 (6.3)	9 (45.0)	0.000	4 (33.3)	4 (33.3)	1.000
FEV1	2.47 ± 0.62	2.72 ± 0.51	0.078	2.48 ± 0.59	2.51 ± 0.54	0.882
FEV1%	81.26 ± 5.44	80.15 ± 4.62	0.410	79.83 ± 3.56	80.00 ± 4.95	0.913
MDL (cm)	2.00 ± 0.86	2.64 ± 1.31	0.053	2.12 ± 0.99	2.32 ± 1.23	0.640
Lesion site			0.178			0.539
Left upper lobe	18 (28.1)	5 (25.0)		5 (41.7)	4 (33.3)	
Left lower lobe	13 (20.3)	2 (10.0)		2 (16.7)	2 (16.7)	
Right upper lobe	18 (28.1)	9 (45.0)		4 (33.3)	4 (33.3)	
Right middle lobe	4 (6.2)	0		0	0	
Right lower lobe	11 (17.2)	4 (20.0)		1 (8.3)	2 (16.7)	
Pathological type			0.097			0.574
SQC	12 (18.8)	9 (45.0)		4 (33.3)	5 (41.7)	
ADC	49 (76.6)	11 (55)		7 (58.3)	7 (58.3)	
Others	3 (4.7)	0		1 (8.3)	0	
Operation style			1.000			1.000
Lobectomy	55 (85.9)	17 (85.0)		10 (83.3)	10 (83.3)	
Sublobar resection	9 (14.1)	3 (15.0)		2 (16.7)	2 (16.7)	
Pathological stage			0.008			0.871
IA	44 (68.8)	9 (45.0)		7 (58.3)	7 (58.3)	
IB	11 (17.2)	1 (5.0)		2 (16.7)	1 (8.3)	
IIA	2 (3.1)	1 (5.0)		0	1 (8.3)	
IIB	3 (4.7)	1 (5.0)		1 (8.3)	0	
IIIA	4 (6.3)	8 (40.0)		2 (16.7)	3 (25.0)	

COPD, Chronic obstructive pulmonary disease; MDL, Maximum diameter of lesion; SQC, Squamous carcinoma; ADC, Adenocarcinoma.

**Table 2:** Perioperative outcome between the two matched groups.

**Variables**	**Conservative treatment group *N* (%) (*n* = 12)**	**Chest tube group *N* (%) (*n* = 12)**	***P*-value**
Operation time (min)	101.67 ± 11.46	98.33 ± 5.29	0.386
Blood loss (mL)	53.33 ± 8.87	63.33 ± 14.35	0.104
Pleural adhesion	6 (50.0)	3 (25.0)	0.453
Average hospital stay time (d)	9.75 ± 1.60	8.00 ± 1.12	0.003
Chest tube removal time (d)	8.58 ± 1.67	6.92 ± 1.08	0.005
Thoracic drainage volume (mL)	844.58 ± 242.44	897.50 ± 266.53	0.660
Visual analog scale (VAS)	1.00 ± 0.01	1.58 ± 0.58	0.020
Serum C-reactive protein (mg/L)	39.80 ± 22.95	53.29 ± 28.23	0.299

## Discussion

VATS is currently considered the primary treatment modality for early-stage NSCLC (3). With advancements in technology, uniportal VATS has become the mainstream surgical technique due to its advantages of minimal invasiveness, rapid recovery, and less pain. Most importantly, compared with conventional thoracoscopic surgery, the oncological prognosis is almost the same (12). However, postoperative complications remain unchanged, with PAL continuing to be one of the most common postoperative complications in thoracic surgery, clinically manifested as pneumothorax. PAL has numerous adverse effects on patients, significantly prolonging the duration of chest tube drainage and hospitalization (9, 13). It can also lead to severe complications such as extensive subcutaneous emphysema, respiratory distress, pulmonary infection, wound infection, and empyema, thereby increasing the psychological and economic burden on patients and contradicting the principles of ERAS.

There are numerous factors contributing to the occurrence of PAL, with alveolar air leakage due to visceral pleural rupture from surgical trauma being the primary cause. Rivera et al. demonstrated that surgical factors can influence the occurrence of PAL (13). In most cases, PAL results from the dissection of visceral pleural adhesions or the presence of incomplete development of pulmonary fissures (14, 15). For our cases, several challenges are presented by the uniportal VATS approach with PAL: Firstly, uniportal VATS makes it difficult to separate adhesions at the base of the thoracic cavity and around the operative uniport, increasing the likelihood of visceral pleural damage and air leakage. Secondly, the incomplete development of pulmonary fissures or unclear anatomical structures of the lung parenchyma led to substantial visceral pleural damage, further increasing the risk of air leakage. Lastly, the common practice is not creating a separate chest tube insertion incision after uniportal VATS. Usually, we placed the tube in a higher placement because of the original incision. Thus, the tube is insufficient for draining air and fluid from the lower thoracic cavity, thereby contributing to poor drainage. To address this issue, we retrospectively evaluated this specific surgical approach, comparing outcomes before and after the chest tube placement. To obtain more reliable comparisons, we employed the Propensity Score Matching (PSM) method to balance key variables and mitigate selection bias between the groups.

The chest tube plays a crucial role in managing postoperative PAL (16, 17). Regarding chest tube management, the majority of experts in previous literature advocate for single-tube placement as the first choice. You et al. thinks that compared with double chest drains, single chest drain has its advantages and safety for pulmonary lobectomy (18). However, our observations have led us to identify three phenomena: Firstly, a higher incidence of postoperative PLA following lobectomy, possibly due to the larger residual pleural cavity; Secondly, PLA patients with subcutaneous emphysema generally have poor lung quality, and the original chest tube cannot provide sufficient air drainage. Extra air can penetrate the muscles of the wound and form subcutaneous emphysema. This phenomenon usually requires additional catheterization because a single chest tube is not enough to meet their clinical needs. Finally, the positioning of a single chest tube may not align with the ideal location to some patients, leading to suboptimal drainage of the upper thoracic cavity. This misalignment may be due to changes in chest tube positioning during wound closure, body position changes, or postoperatively expanded lung tissue compressing and displacing the chest tube. For all these cases, another chest tube may need to be placed to increase effective drainage (19). We reviewed previous cases and traditional textbooks and found that double chest tubes may be beneficial for these patients.

The choice of treatment modality for PLA primarily depends on its efficacy and feasibility. Our standard approach for treating most cases of PLA involves thoracic negative pressure suction and pleurodesis with agents such as diluted povidone-iodine, autologous blood, or 50% glucose solution (20–22). Most cases can solve with these methods. According to our experience, we need to keep the patient completely supine in bed when we perform this type of chemical pleurodesis. Inject the drug through the chest tube and instruct the patient to cough to expel the remaining air in the chest, and make the highest point of the chest tube higher than the patient's chest plane, ensure the air can flow out and the fluid can be fully retained in the chest. This method promotes adhesions in the roof of the pleural cavity. We do not recommend Trendelenburg position as it may cause symptoms such as dizziness or hypotension etc. Other methods such as the use of digital chest drainage systems have also been reported in other literature. Comacchio et al. believe that the use of digital drainage systems can remove chest drains earlier than traditional systems (23). Bao et al. deems that discharge patient' chest tube management can be accomplished in selected patients without a major increase in morbidity or mortality (24). However, these methods cannot be implemented in primary hospitals due to technical feasibility.

Chest tube placement is more appropriate for patients with significantly larger residual cavities, incomplete lung re-expansion with subcutaneous emphysema, and prolapsed chest tubes (25). These patients share common clinical characteristics, including inadequate air drainage and failure of the visceral and parietal pleura to adhere, resulting in persistent pneumothorax. In our clinical practice, we usually take chest X-rays routinely on the 1st and 3rd day after VATS surgery. For the situations mentioned above (25), including situations I and II, we will perform extracorporeal negative pressure drainage. If the situation does not improve, we will immediately insert the chest tube because in these cases, a single chest tube is not sufficient to remove the excess gas. While for situation III (the grade air leak does not decrease after conservative treatment), we decide whether to intubate based on the degree of air leak after conservative treatment of the patient. Typically, we start conservative management on the 3rd postoperative day for patients with air leak. Pleural adhesion agents such as diluted povidone-iodine are injected intrathoracically once a day for two consecutive times. If the air leak does not decrease, we will insert the chest tube decisively. Patients who require chest tube placement have grade II or III air leaks, because grade I air leaks will almost always improve after conservative treatment.

We must carefully consider the appropriateness of chest tube placement because of the inherent risks of this action. Remember, conservative treatment is always the first choice for patients with PLA because it is less invasive and has lower risks. In most cases of group B, postoperative lungs are not fully collapsed, and improper handling may cause secondary harm to the patient, leading to various short-term complications, including increased pain, pulmonary contusion, hemothorax, arrhythmias such as atrial fibrillation, and even the need for secondary repair surgery (26). In our experience, seven patients experienced minor complications. The prevalence of increased pain was the most frequently observed finding, aligning with the results of our study (1.58 ± 0.58 vs. 1.00 ± 0.01, *P* = 0.020). Four patients received additional analgesic drugs. Another patient had atrial fibrillation after placement that was controlled by antiarrhythmic therapy. One patient had little thoracic hemorrhagic exudation after the operation, which was promptly treated with hemostatic drugs. The final patient experienced lung damage due to a puncture caused by the needle, which exacerbated the air leak, and received a secondary surgical intervention. In order to avoid the occurrence of the above complications, we have returned to the traditional double chest tube placement method in recent surgeries for patients with poor lung quality. Our experience with chest tube placement has yielded several insights, and the procedural steps are illustrated in the accompanying (Figure 4). The primary technical points involve creating an artificial pneumothorax and utilizing the needle to guide the placement of the chest tube at a high thoracic position. For surgeons with limited experience, we advocate for more stringent preoperative assessment and more cautious intraoperative decision-making as essential factors to ensure the safety of this challenging procedure.

Our study indicates that although chest tube repositioning may temporarily increase patient discomfort and burden, it remains a simple and safe technique. It is also much less cost-effective than endoscopic treatment (27, 28). After matching patients in the early stages, compared to the conservative treatment group, chest tube repositioning significantly reduced hospitalization time and financial burden. In terms of short-term outcomes, there was no significant increase in thoracic drainage volume (844.58 ± 242.44 vs. 897.50 ± 266.53 ml, *P* = 0.660), patients showed lung recovery well without infection before discharge (Serum C-reactive protein: 39.80 ± 22.95 vs. 53.29 ± 28.23 mg/L, *P* = 0.299), and there were no records of readmission at 1 month postoperatively.

The limitations of our study are evident. Due to the retrospective nature of the study and the relatively small sample size, selection bias is inevitable. Additionally, given the limited sample size, the long-term outcomes remain to be further reviewed. Future studies with larger sample sizes or prospective randomized controlled study are necessary to validate the potential benefits of additional chest tube placement.

## Conclusion

Based on our study, additional chest tube placement appears to be safe and effective and may serve as a suitable alternative in selected patients with prolong air leaks. Our findings must be confirmed by large-sample, prospective randomized controlled studies.

## Data Availability

The original contributions presented in the study are included in the article/supplementary material, further inquiries can be directed to the corresponding author.

## References

[1] Bray F Laversanne M Sung H Ferlay J Siegel RL Soerjomataram I. Global cancer statistics 2022: GLOBOCAN estimates of incidence and mortality worldwide for 36 cancers in 185 countries. CA Cancer J Clin. (2024) 74:229–63. 10.3322/caac.21834 38572751

[2] Zheng RS Chen R Han BF Wang SM Li L Sun KX. Cancer incidence and mortality in China, 2022. Zhonghua Zhong Liu Za Zhi. (2024) 46:221–31. 10.3760/cma.j.cn112152-20240119-00035 38468501

[3] Yang HX Woo KM Sima CS Bains MS Adusumilli PS Huang J. Long-term survival based on the surgical approach to lobectomy for clinical stage I nonsmall cell lung cancer: comparison of robotic, video-assisted thoracic surgery, and thoracotomy lobectomy. Ann Surg. (2017) 265:431–7. 10.1097/SLA.0000000000001708 28059973 PMC5033685

[4] Scarci M Gonzalez-Rivas D Schmidt J Bedetti B. Management of intraoperative difficulties during uniportal video-assisted thoracoscopic surgery. Thorac Surg Clin. (2017) 27:339–46. 10.1016/j.thorsurg.2017.06.002 28962706

[5] Ponholzer F Ng C Maier H Lucciarini P Öfner D Augustin F. Risk factors, complications and costs of prolonged air leak after video-assisted thoracoscopic surgery for primary lung cancer. J Thorac Dis. (2023) 15:866–77. 10.21037/jtd-21-2011 36910082 PMC9992586

[6] Lapidot M Levy Faber D Bueno R. Prolonged air leak after lung surgery-prevalent complication without a perfect solution. J Thorac Dis. (2023) 15:5285–6. 10.21037/jtd-23-1180 37969302 PMC10636445

[7] Hoeijmakers F Hartemink KJ Verhagen AF Steup WH Marra E Röell WFB. Variation in incidence, prevention and treatment of persistent air leak after lung cancer surgery. Eur J Cardiothorac Surg. (2021) 61:110–7. 10.1093/ejcts/ezab376 34410339 PMC8715850

[8] Bertolaccini L Brunelli A. Devising the guidelines: the techniques of uniportal video-assisted thoracic surgery-postoperative management and enhanced recovery after surgery. J Thorac Dis. (2019) 11:S2069–72. 10.21037/jtd.2019.01.62 31637040 PMC6783708

[9] Attaar A Luketich JD Schuchert MJ Winger DG Sarkaria IS Nason KS. Prolonged air leak after pulmonary resection increases risk of noncardiac complications, readmission, and delayed hospital discharge: a propensity score-adjusted analysis. Ann Surg. (2021) 273:163–72. 10.1097/SLA.0000000000003191 30829700

[10] Seder CW Basu S Ramsay T Rocco G Blackmon S Liptay MJ. A prolonged air leak score for lung cancer resection: an analysis of the society of thoracic surgeons general thoracic surgery database. Ann Thorac Surg. (2019) 108:1478–83. 10.1016/j.athoracsur.2019.05.069 31323209 PMC8693718

[11] Mohrsen S McMahon N Corfield A McKee S. Complications associated with pre-hospital open thoracostomies: a rapid review. Scand J Trauma Resusc Emerg Med. (2021) 29:166. 10.1186/s13049-021-00976-1 34863280 PMC8643006

[12] Shen Y Wang H Feng M Xi Y Tan L Wang Q. Single- versus multiple-port thoracoscopic lobectomy for lung cancer: a propensity-matched study. Eur J Cardiothorac Surg. (2016) 49(Suppl.1):i48–53. 10.1093/ejcts/ezv358 26464451

[13] Rivera C Bernard A Falcoz P-E Thomas P Schmidt A Bénard S. Characterization and prediction of prolonged air leak after pulmonary resection: a nationwide study setting up the index of prolonged air leak. Ann Thorac Surg. (2011) 92:1062–8. 10.1016/j.athoracsur.2011.04.033 21871301

[14] Stamenovic D Bostanci K Messerschmidt A Jahn T Schneider T. Fissureless fissure-last video-assisted thoracoscopic lobectomy for all lung lobes: a better alternative to decrease the incidence of prolonged air leak? Eur J Cardiothorac Surg. (2016) 50:118–23. 10.1093/ejcts/ezv455 26792925

[15] Aprile V Bacchin D Calabrò F Korasidis S Mastromarino MG Ambrogi MC. Intraoperative prevention and conservative management of postoperative prolonged air leak after lung resection: a systematic review. J Thorac Dis. (2023) 15:878–92. 10.21037/jtd-22-736 36910073 PMC9992588

[16] Yan S Wang X Wang Y Lv C Wang Y Wang J. Intermittent chest tube clamping may shorten chest tube drainage and postoperative hospital stay after lung cancer surgery: a propensity score matching analysis. J Thorac Dis. (2017) 9:5061–7. 10.21037/jtd.2017.11.08 29312711 PMC5757041

[17] Deng B Qian K Zhou J-H Tan Q-Y Wang R-W. Optimization of chest tube management to expedite rehabilitation of lung cancer patients after video-assisted thoracic surgery: a meta-analysis and systematic review. World J Surg. (2017) 41:2039–45. 10.1007/s00268-017-3975-x 28289835

[18] You J Zhang H Li W Dai N Zheng Z. Single versus double chest drains after pulmonary lobectomy: a systematic review and meta-analysis. World J Surg Oncol. (2020) 18:175. 10.1186/s12957-020-01945-1 32690055 PMC7372892

[19] Tanaka M Sagawa M Usuda K Machida Y Ueno M Motono N. Postoperative drainage with one chest tube is appropriate for pulmonary lobectomy: a randomized trial. Tohoku J Exp Med. (2014) 232:55–61. 10.1620/tjem.232.55 24492628

[20] Park JB Lee SA Lee WS Kim YH Hwang JJ. The management of chemical pleurodesis with viscum album in patients with persistent air leakage. J Thorac Dis. (2018) 10:371–6. 10.21037/jtd.2017.12.67 29600069 PMC5863110

[21] Chaari Z Hentati A Ayed AB Abid W Frikha I. Effectiveness and safety of povidone iodine for prolonged lung air-leak after lung surgery. Asian Cardiovasc Thorac Ann. (2022) 30:314–20. 10.1177/02184923211067637 34904450

[22] Hugen N Hekma EJ Claessens NJM Smit HJM Reijnen MMPJ. Efficacy of an autologous blood patch for prolonged air leak: a systematic review. Ann Thorac Surg. (2022) 114:1064–71. 10.1016/j.athoracsur.2021.05.047 34115999

[23] Comacchio G Marulli G Mendogni P Andriolo LG Guerrera F Brascia D. Comparison between electronic and traditional chest drainage systems: a multicenter randomized study. Ann Thorac Surg. (2023) 116:104–9. 10.1016/j.athoracsur.2023.02.057 36935028

[24] Bao F Dimitrovska NT Hu S Chu X Li W. Safety of early discharge with a chest tube after pulmonary segmentectomy. Eur J Cardiothorac Surg. (2020) 58:613–8. 10.1093/ejcts/ezaa097 32236542

[25] Satoh Y. Management of chest drainage tubes after lung surgery. Gen Thorac Cardiovasc Surg. (2016) 64:305–8. 10.1007/s11748-016-0646-z 27048219

[26] Anderson D Chen SA Godoy LA Brown LM Cooke DT. Comprehensive review of chest tube management: a review. J Am Med Assoc Surg. (2022) 157:269–74. 10.1001/jamasurg.2021.7050 35080596

[27] Abu-Hijleh M Styrvoky K Anand V Woll F Yarmus L Machuzak MS. Intrabronchial valves for air leaks after lobectomy, segmentectomy, and lung volume reduction surgery. Lung. (2019) 197:627–33. 10.1007/s00408-019-00268-7 31463549

[28] Konagaya K Yamamoto H Nishida T Morita T Suda T Isogai J. Negative-pressure wound therapy to treat thoracic empyema with COVID-19-related persistent air leaks: a case report. Front Med. (2022) 9:970239. 10.3389/fmed.2022.970239 36035387 PMC9402970

